# Evaluation of auditory processing before and after treatment in patients with speech disorders

**DOI:** 10.1590/S1808-86942010000500024

**Published:** 2015-10-22

**Authors:** Tiago Mendonça Attoni, Victor Gandra Quintas, Alexandre Hundertmarck Lessa, Carolina Lisbôa Mezzomo, Helena Bolli Mota

**Affiliations:** 1 MSc Student in Human Communication Disorders – Federal University of Santa Maria, Speech and Hearing Therapist; 2 MSc Student in Human Communication Disorders – Federal University of Santa Maria, Speech and Hearing Therapist; 3 Speech and Hearing Therapy Student – Federal University of Santa Maria - UFSM, Santa Maria, RS; 4 PhD in Applied Linguistics; Speech and Hearing Therapist; Professor at the Graduate Program in Human Communication Disorders - Federal University of Santa Maria - UFSM, Santa Maria, RS; 5 PhD in Applied Linguistics; Speech and Hearing Therapist; Professor at the Graduate Program in Human Communication Disorders - Federal University of Santa Maria - UFSM, Santa Maria, RS

**Keywords:** hearing, child, language therapy

## INTRODUCTION

Speech changes are characterized as abnormal manifestations of spoken language patterns. Children with such changes use speech rules which are far from the adult-target speech in unknown etiological circumstances. One of the factors which can be associated with this speech abnormality is auditory processing^1^ which is described as changed in these cases^2^,^3^.

We, hereby, present a case report and a review of the pertaining literature.

## CASE PRESENTATION

AGFA, a 7-year old boy, came to the Speech Therapy Service, having been referred from school, with the complaint of having speech changes. Today, he is in the 2nd year (first grade) and has good school performance.

As to the gestational period, there were no complications, the mother was 43 years when she discovered she was five months pregnant. In the beginning, she was upset and nervous, but she accepted the pregnancy. The delivery was natural, pre-term, at about 32 weeks. The newborn weighed 2.400g, and was 48 cm tall.

In the speech assessment, speech sound changes were confirmed. The following phonemes were changed in speech: /f/, /v/, /s/, /Z/, /S/, /r/, /l/, /'/, /R/ and an absence of all combined consonants.

Upon tonal and vocal audiologic evaluation, the results were within normal standards. We found a type A tympanometric curve and altered acoustic reflexes in all the frequencies.

In order to test the auditory processing we used the Digits Dichotic and SSW tests. The results of auditory processing in acoustic signal decoding and organization were altered, as were selective attention skills, memory and auditory analysis-synthesis tests.

Speech and hearing therapy aimed at promoting auditory, tactile and visual stimulation by means of the Modified Cycles Model. There were 15 sessions planned for the semester, and the following speech and hearing processes were worked upon: affricates (phonemes /Z/ and /S/), liquid substitution (phonemes /r/ and /'/) and reduction of consonant meetings (meetings with /l/ and with /r/).

At the end of the semester there was a new speech and auditory processing evaluation. Upon speech reassessment, the patients showed a significant improvement on the following phonemes: /r/, /l/ e /'/, besides the consonant combinations with /l/. In the auditory processing reassessment, the results from both dichotic tests were within normal limits, excluding any type of change.

## DISCUSSION

The speech and hearing changes still bears an idiopathic cause. The association found with auditory processing skills has opened new horizons for studies; nonetheless the use of such investigative methodology is recent. The results from the dichotic test in the present study confirm the relationship between these speech and auditory processing changes^2^.

The use of a therapy model based on auditory, visual and tactile clues, besides benefiting the child to acquire new speech sounds, seems to also help in auditory processing. This speech therapeutic model had similar success to the therapy which uses auditory training for cases of auditory processing changes^4^,^5^.

**Results from the auditory processing before and after therapy for speech changes.**

**Table:** 

	SSW Percentage of errors		Digits Dichotic in correct answers *	
Stimulus condition	CR CL	FA	AT	AL
Pre-therapy evaluation	70,0 75,0	36	11	10
Post Therapy evaluation	10,0 12,5	75	37	34

CR= competitive right; CL= competitive left; FA= free attention; RA= Attention to the right; AL= attention to the left.

* Gross values.

Without any doubts, the child recovered the skill to deal with acoustic information. A learning process for new sounds generated a neural plasticity, showing improvements in auditory processing functions and skills.

The success in speech and hearing therapies and neural plasticity are closely linked, because if there is training about auditory systematic, there will be an improvement in its functioning^6^.

## FINAL REMARKS

It is important to investigate the auditory processing in children with speech impairment, since these can be associated. We also stress that the speech therapy model (modified cycles) caused positive changes to speech performance and on the functioning of the auditory processing.
